# Salecan-Clay Based Polymer Nanocomposites for Chemotherapeutic Drug Delivery Systems; Characterization and In Vitro Biocompatibility Studies

**DOI:** 10.3390/ma13235389

**Published:** 2020-11-27

**Authors:** Paula Ecaterina Florian, Madalina Icriverzi, Claudia Mihaela Ninciuleanu, Elvira Alexandrescu, Bogdan Trica, Silviu Preda, Raluca Ianchis, Anca Roseanu

**Affiliations:** 1 Department of Ligand-Receptor Interaction, Institute of Biochemistry of the Romanian Academy, Splaiul Independentei 296, 060031 Bucharest, Romania; florian_paula@yahoo.com (P.E.F.); radu_mada@yahoo.co.uk (M.I.); 2 National R&D Institute for Chemistry and Petrochemistry ICECHIM—Bucharest, Splaiul Independentei 202, 6th District, P.O. Box 35/174, 0600021 Bucharest, Romania; claudia.ninciuleanu@yahoo.com (C.M.N.); elviraalexandrescu@yahoo.com (E.A.); trica.bogdan@gmail.com (B.T.); 3 Institute of Physical Chemistry “Ilie Murgulescu”, Romanian Academy, Spl. Independentei 202, 6th District, P.O. Box 194, 060021 Bucharest, Romania; predas01@yahoo.co.uk

**Keywords:** nanocomposites, Salecan, clay, doxorubicin

## Abstract

Salecan is a microbial polysaccharide suitable to obtain hydrogel for biomedical applications due to the excellent hydrophilicity and biocompatibility properties. In this work, Salecan of different concentrations was introduced into polymethacrylic acid (PMAA) in the presence of clay to form novel semi synthetic hydrogel nanocomposites systems and loaded afterwards with doxorubicin (DOX). The physical–chemical characteristics of the nanocomposites systems and their effect on the viability, and morphology of MDBK (Madin–Darby bovine kidney), HT-29 human colorectal adenocarcinoma and Colo 205 human colon adenocarcinoma cell lines were investigated. DOX release from the nanocomposite systems, cell up-take and subsequent effect on cell proliferation was also analyzed. It was found that Salecan concentration determined the swelling behavior, structural parameters and morphological features of the nanocomposite systems. The hydrogen bonds strongly influenced the formation of PMAA–Salecan–clay systems, each component bringing its own contribution, thus demonstrating the achievement of an advanced crosslinked network and a more compacted hydrogel nanocomposite morphology. All the synthesized nanocomposites had negligible toxicity to normal MDBK cells and chemoresistent HT-29 cell line, whereas in the case of Colo 205 cells a decrease by 40% of the cell viability was obtained for the sample containing the highest amount of Salecan. This effect was correlated with the lowest pore size distribution leading to highest available specific surface area and entrapped amount of DOX which was further released from the nanocomposite sample. Corroborating all the data it can be suggested that the synthesized nanocomposites with Salecan and clay could be good candidates as vehicles for chemotherapeutic agents.

## 1. Introduction

To improve efficiency and specificity of anti-tumoral drug delivery while reducing therapeutic drugs side effects and toxicity, encapsulation of chemotherapeutic compounds represents an attractive approach. Additionally, by encapsulating active compound in nanocomposites, the desideration in nanomedicine is to reduce the drug toxicity in normal host tissues and increase its bioactive effect in tumor tissue. 

Incorporation of nanoclays into polymeric complex networks as hydrogels may create promising systems with enhanced capacity of drug retaining and releasing. Hydrogels are three dimensional structures of crosslinked macromolecules, capable of holding high water content or physiological solutions while maintaining their undissolved original structure [1,2,3,4]. However, in swollen state, hydrogels present weak mechanical properties restricting their widespread application. This problem has been addressed over the years by several methods, such as for example to add to hydrogel formulation inorganic nanoparticles [5,6]. By adding clay as inorganic component, hydrogel nanocomposites acquired improved features of principally-enhanced and thermo-mechanical properties, increased swelling capacity, and water permeability. Moreover, their great specific surface and charging make clay nanoparticles able to adsorb bioactive molecules and thus to increase their stability [7,8,9,10]. Several studies proved that the encapsulation of clay layers into hydrogels influenced the burst release phenomena and induced a long term sustained drug delivery. These findings are closely linked with the dispersion degree of the silicate sheet inside the macromolecular crosslinked system as well as to the silicate type and its hydrophobic–hydrophilic features [5,8,11]. 

Another method of counteracting the weak mechanical features of hydrogels, especially natural biopolymer-based hydrogels, is by adding a second polymer network. Thus, synthetic polymer chains which are generally more rigid, can be crosslinked within natural hydrogels, resulting in an interpenetrated network of rigid/flexible polymer chains, where the favorable properties of each polymeric component can be combined to achieve the desired characteristics for specific application [3,12,13]. In this respect, natural biopolymers such as polysaccharides represent suitable candidates for the synthesis of semisynthetic hydrogels for biomedical application due to their very good biocompatibility and availability. Among them, Salecan, a polysaccharide produced by a microorganism namely, *Agrobacterium sp.*, was demonstrated to be very attractive compound for biomaterials fabrication considering its biological but also physical–chemical properties, such as hydrophilic nature, high viscosity, pseudo-plasticity, biocompatibility, and biodegrability. Being obtained from renewable sources with high production rates and simple purification conditions in microorganisms, Salecan may display also economic advantages when used in combination with synthetic polymers. Moreover, the morphology and mechanical strength of several semisynthetic hydrogels containing Salecan were precisely designed by using different Salecan amounts during the synthesis process [14].

In our previous works, Salecan was interpenetrated with polymethacrylic acid chains, a pH sensitive synthetic polymer, to obtain new semisynthetic hydrogel-clay nanocomposites. The biopolymer linear chains were physically entrapped between cross-linked polymer chains in the presence of clay inorganic filler. It was shown that the concentration of the biopolymer and the type of clay influenced the final nanocomposites structure and properties [15,16,17,18]. 

The nanocomposite hydrogel prove to be a suitable formulation when controlled release of the is required and the hydrogel should reach the target unaltered.

Thus, the dynamic mechanical tests revealed higher values of storage modulus and stiffness registered for the previously reported semi-interpenetrated Salecan nanocomposite systems against those obtained for Salecan free hydrogel nanocomposites. This beneficial mechanical effect brought by Salecan was attributed to its elastic properties but also to its OH groups’ interactions with the OH groups from clay surface. Moreover, inorganic filler addition led also to stronger hydrogels as indicated by the increasing values of the storage module with frequency and time. As a result of the intermolecular forces between the silicate and the polymeric networks, the nanocomposites behaved like an elastic solid being able to store energy and remain intact when subjected to stress [13,16].

Considering these findings, our aim was to investigate the potential of pH sensitive hydrogel based nanocomposites to post-encapsulate a chemotherapeutic drug intended to be further released to cancer cells from gastrointestinal tract. Doxorubicin (DOX) is a strong chemotherapeutic agent used in colon cancer and various types of drug delivery systems such as polymeric nanoparticles and conjugates, micelles, exosomes, and paramagnetic nanoparticles have been developed and evaluated for their ability to release DOX at the tumor site [18,19,20,21,22]. Although injectable hydrogels containing DOX for local delivery have been reported [23,24], hydrogel for oral dosage forms are the most preferred delivery route for colon-specific delivery due to their convenience, greater degree of flexibility in manufacturing, improved patient adherence and relatively safe administration [25]. All these findings reveal the need for continuous optimization of hydrogel formula as a vehicle targeted drug delivery.

Thus, our present study mainly focuses on the in vitro biocompatibility of Salecan–PMMA–clay hydrogel nanocomposite previously described [16] loaded with doxorubicin was analyzed. Its effect on the viability, proliferation and morphology of MDBK (Madin–Darby bovine kidney), HT-29 human colorectal adenocarcinoma and Colo 205 human colon adenocarcinoma cell lines was evaluated using colorimetric method and fluorescence microscopy. DOX release from the nanocomposite hydrogel, cell up-take, and subsequent effect on tumor proliferation was investigated by immunofluorescence microscopy and flow cytometry. 

## 2. Material and Methods

### 2.1. Reagents

The monomer—(methacrylic acid from Janssen Chimica), biopolymer—Salecan (Suzhou Chemicals, Suzhou, China), crosslinker—N, N-methylenebisacrylamide (Sigma Aldrich, St. Louis, MO, USA), initiator—ammonium persulfate (Sigma Aldrich, St. Louis, MO, USA) and drug—DOX (doxorubicin hydrochloride, Cayman Chemical, Ann Arbor, MI, USA) were used as received. Commercial clay-Cl 93A (Southern Clay Products Inc., Gonzales, TX, USA) is an organomodified clay with ammonium salts of fatty acids (methyl, tallow, bis-2-hidroxyethyl (methyl, dehydrogenated tallow)-(Cloisite^®^ 93A, 90 meg/100 g)), was used without further purification. 

### 2.2. Hydrogel Nanocomposites Preparation

The hydrogel composites were synthesized the polymerization process of methacrylic acid in the presence of Salecan and commercial clay-Cl 93A. Clay amount was maintained constant with respect to the monomer-methacrylic acid, while varying biopolymer concentration. In order to assure a good swellability together with improved mechanical properties and based on the previously published results [15,16,17], the concentration of clay was selected to be 10% *w/v* in monomer; ~1% from the total mass and was kept constant for all the hydrogel nanocomposites samples. Thus, four systems were synthesized (Table 1) and their physical–chemical-biological features were followed. 

The preparation of semi-synthetic nanocomposites systems was described elsewhere and adjusted to specific conditions [16,17]. Briefly, a dispersion of 0.6 g Cl 93A clay in 42 mL of deionized water was obtained through magnetically stirring at ambient temperature. An advanced dispersion was obtained further through the use of an ultrasound probe. Salecan powder (0.06, 0.24, or 0.48 g) was added and the system was exposed again to ultrasonication. Six milliliters of methacrylic acid (MAA) and 6 mL sol. 1% *w/v* N, N’-methylene bis acrylamide (BIS), were introduced in the system under mechanical stirring and nitrogen gas. Further, the dispersion was placed in an ice bath and ultrasonicated. Six milliliters of sol 1.2% *w/v* pf ammonium persulfate (APS) was gently poured and the dispersion. The initial reaction system was introduced by syringe into an artisanal glass mold. The mold that was placed into a water bath with controlled temperature. After 6 h, the obtained hydrogel composite samples were washed for the elimination of residual reactants. No degradation of the samples was registered the samples remaining stable and intact during the washing process with deionized water during 10 days. Cylinder hydrogels were shaped with an eyelet punch and further dried by lyophilization. 

The hydrogels were weighed beforehand (8.5–10 µg) and places in a vial with 1.5 mL of doxorubicin solution (100 µg/mL) at room temperature for 24 h. Subsequently, unabsorbed and superficially adsorbed DOX solution was removed from the swollen hydrogel samples with deionized water. The absorbance at 481 nm of DOX remained in the DOX solution after drug entrapment, was measured using a UV-Vis Thermo Scientific spectrophotometer. The efficiency of DOX loading in the nanocomposite hydrogel samples was determined using the equation:

Efficiency (weight, %) = (W0 − We)/W0 × 100.(1)

W0 and We are the total amounts of DOX in the initial immersion solution and the solution remained after removing the loaded samples. Charging efficiency was calculated as the average of 20 measurements.

### 2.3. Physical–Chemical Analysis

Swelling tests were conducted using the lyophilized samples. The samples were placed in vials in deionized water at 37 °C the swollen samples being weighted at pre-settled times. The swelling degree (SD) was determined by the equation:

SD = (Wh − Wi)/Wi.(2)

Wh is the weight of hydrated sample at the pre-established time and Wi the weight of the dried sample. Swelling degree was calculated as the average of three measurements.

FT-IR spectra were registered on powdered in the range 400–4000 cm^−1^, with Tensor 37 from Bruker, Woodstock, NY, USA in ATR mode.

X-Ray diffractograms were obtained using Rigaku Ultima IV X-ray diffractometer, Tokyo, Japan. The measurements were performed on powdered samples at room temperature. The equipment was set in parallel beam geometry system in two specific configurations for the wide angle and low angle range.

Transmission electron microscopy images were obtained using Tecnai™ G2 F20 TWIN Cryo-TEM instrument from FEI Company™ (Eindhoven, The Netherlands) Thin slices (~100 nm) of epoxy included samples were examined from carbon film grids.

### 2.4. Cell Culture

The MDBK cell line (Madin–Darby bovine kidney) obtained from ECACC (European Collection of Animal Cell Culture, Porton Down, UK), HT-29 human colorectal adenocarcinoma cell line (a kind gift from Dr. Frank-Dietmar Bohmer, Institute of Molecular Cell Biology, Medical Faculty Friedrich-Schiller University, Jena, Germany) and Colo 205 human colon adenocarcinoma cell line (gift from Dr. Ana Calugaru, I. Cantacuzino Institute, Bucharest, Romania) were maintained in RPMI 1640 or DMEM medium respectively (37 °C, 5% CO_2_) supplemented with stable glutamine, 10% fetal bovine serum (FBS) and 100 U/mL penicillin, 100 μg/mL streptomycin (all from Gibco^®^ by Life Technologies, NY, USA). 

Cells with more than 90% viability as determined by trypan blue staining were used in this work (passage nr. 8–10).

### 2.5. Sterilization

For biological in vitro experiments all nanocomposite systems were subjected to sterilization using an ethanol solution 70% in PBS (phosphate buffered saline pH 7.4). After 2 min of treatment the samples were rinsed in PBS three times and finally immersed in culture medium.

### 2.6. DOX Release 

For all experiments involving DOX release, the composite hydrogels previously loaded with Dox (PC, PCS1, PCS2, PCS3) were immersed in 1 mL PBS with pH resembling tumoral (pH = 5.5) and normal physiologic conditions (pH 7.4). All experiments were performed at 37 °C with continuous stirring at 100 rpm. At specific time points 100 μL of supernatant were collected and transferred in a 96 wells plate with opaque walls. The absorbance at 480 nm, which is directly correlated with the level of DOX released into the supernatant, was measured using a FLUOStar Omega spectrofluorimeter (BMG Labtech) equipped with Omega acquisition software v3.00.R2. The volume of supernatant collected for point measurements was returned into the final volume to determine the cumulative DOX release from the nanocomposites systems. The quantity of DOX was calculated by data extrapolation using a standard curve of DOX and reported as percent of initial quantity of DOX in the systems tested. 

### 2.7. Cytotoxicity Assay

5 × 10^4^ cells/well were cultured in 24-well plate for 24 h before addition of hydrogel systems. Then, the cells were incubated with nanocomposite hydrogels for 30 min and 3 h. After these time points cell viability was determined by MTS ([3-(4, 5-dimethylthiazol-2-yl)-5-(3-carboxymethoxyphenyl)-2-(4-sulfophenyl)-2H-tetrazolium, inner salt]) colorimetric method (CellTiter 96^®^ Aqueous Non-Radioactive Cell Proliferation Assay, Promega, Fitchburg, WI, USA) colorimetric method. Briefly, a mixture of cell culture medium and MTS compound was added to each well for 30 min, then 100 μL of solution was removed, transferred to a 96-well plate clear bottom (Nunc, Thermo Fisher Scientific, CA, USA) for optical measurement at 450 nm (Mithras LB 940 DLReady, Berthold, Bad Wildbad, Germany). Cell viability was expressed as percent of control (untreated cells).

### 2.8. Immunofluorescence Microscopy

MDBK, HT-29 and Colo 205 cells grown for 24 h on coverslip in 24 wells plate (5 × 10^4^ cells/well) were treated with the nanocomposites systems, 30 min and 3 h at 37 °C. Cells washed with PBS, fixed for 10 min with p-formaldehyde (4%) were permeabilized with 0.2% Triton-X-100 (3 min at room temperature-RT). Unspecific sites were blocked for 1 h with 0.5% bovine serum albumin (BSA) solution in PBS. Cells were labelled with anti-Ki67 antibodies (RM-9106-S SP6, Thermo Fisher Scientific, CA, USA) dil. 1:100 in 0.5% PBS-BSA) for 30 min at RT and then for 1 h with anti-rabbit Alexa-Fluor 488 antibodies (R37116 ready-to-use, Invitrogen, Thermo Fisher Scientific, CA, USA). The nuclei were counterstained with Hoechst fluorescent dye (blue) (dil. 1:3000 in PBS, H 21492) for 1 min at RT. The negative control (Ki67- absent) was obtained by treating the cells with 70% ethylic alcohol (3 min) while untreated cells represent the positive control (Ki67+).

ProLong Gold antifade (Molecular Probes, Life Technologies, Eugene, OR, USA) was used for samples mounting on microscope slide DOX internalization (red) was visualized using 40× lens of Zeiss Axiocam ERc5s Apotom microscope (Jena, Germany) with ApoTome.2 cursor mode and AxioVision4.8 software (Zeiss, Jena, Germany).

### 2.9. Flow Cytometry

One day before the experiment, MDBK, HT-29 sand Colo 205 cells were seeded in 24 wells plate (80% confluence). Next day cells were incubated with hydrogels systems for 30 min or 3 h at 37 °C, and after the incubation time washed with PBS buffer to remove unbound DOX. Cells were trypsinized (Trypsin-EDTA 0.05%), fixed with 4% PFA (10 min, RT), washed and resuspended in FACS buffer (1 × 10^6^ cells/mL in 2% BSA-PBS). Acquisition was carried out on 10,000 events using BD FACSCalibur™ (Becton Dickinson, NJ, USA) cytometer and was restricted to live-dead discrimination on forward scatter/side scatter (FSC/SSC) dot plot to exclude dead cells. Specific Dox internalization was evaluated as a median fluorescence intensity using BD FACS CellQuestPro (Becton Dickinson, NY, USA). Data were exported and analyzed using the web-based application Cytobank [26]. 

### 2.10. Statistical Analysis

Results were expressed as an average ± S.D of values obtained from triplicate samples were analyzed with GraphPad Prism software (version 5; La Jolla, CA, USA) using one-way ANOVA with Tukey’s Multiple Comparison test or two-way ANOVA. *p*-value < 0.05 are consider statistically significant. 

## 3. Results and Discussions 

Physical–chemical and biological properties, including structural parameters, swelling behavior, morphological features, cell viability, proliferation, and internalization are the key factors which determine hydrogels further applications.

### 3.1. Nanocomposite Hydrogels Characterization

**Figure 1:**
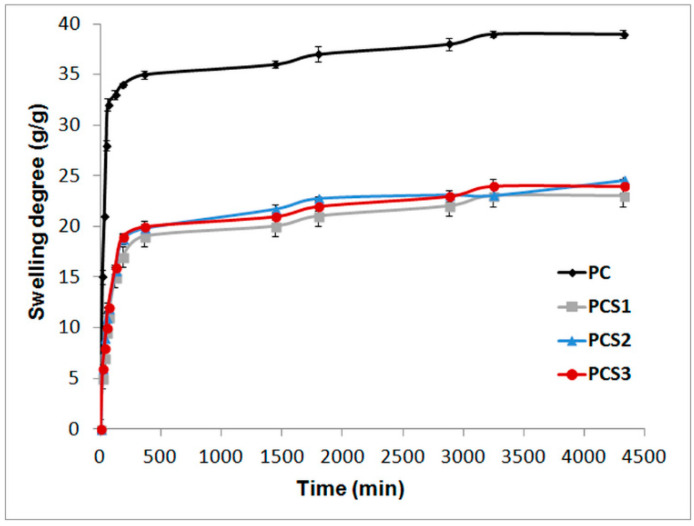
Swelling profiles of the composite samples.

*Swelling properties*. Chemically crosslinked hydrogels were synthesized in water proved swelling-resistant behavior conserving their shape without dissolution for more than 1 year. The swelling behavior of the composite samples throughout 72 h is displayed in Figure 1. During 180 min the composite hydrogels swelled rapidly and reached almost the equilibrium state in 360 min. After that, the samples retained less water following a semi-constant plateau. A noticeable diminution of swelling degree was determined for all Salecan-containing composite samples, compared to the blank composite sample. Obviously, the substitution of the synthetic polymer and clay with Salecan, despite concentration, does not equal their initial contribution to the degree of swelling. At a pH of 5.4, the carboxyl groups in the polymethacrylic acid network undergo an ionization process, the polymethacrylic acid chains being negatively charged, thus influencing the swelling degree [27,28]. The number of carboxylic groups from the synthetic polymer in the semi-interpenetrating network decreases with the addition of Salecan. Moreover, a reaction of condensation between the COOH groups specific to polymethacrylic acid and the OH groups of the biopolymer may occur, leading to a decrease in swelling for Salecan containing composites. 

This dropdown behavior may be explained also by the formation of supplementary physical/chemical crosslinks between the components with consequences on their morphological features and further swelling behavior. Salecan, but also clay, were demonstrated earlier to function as physical/chemical crosslinking agents owing to their OH groups, thus leading to the generation of additional crosslinking points [29,30,31,32]. Thereby, the mobility of polymer networks was restricted with direct implications on the hydrogel porosity. Low network porosity caused lessened absorption of water molecules, consequently, the decrease of the swelling degree.

**Figure 2:**
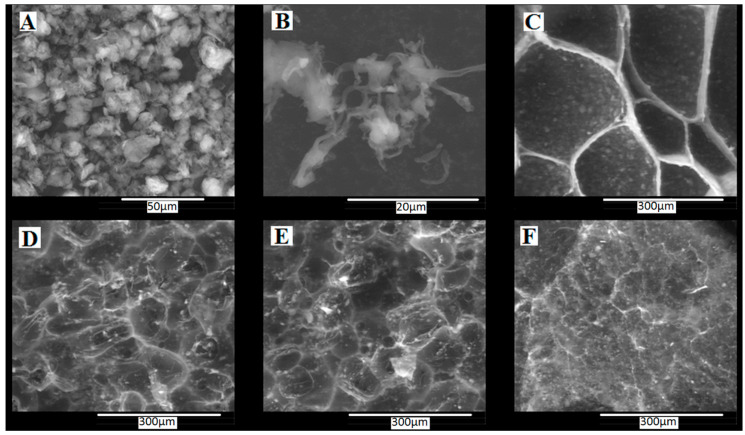
SEM images showing the morphology of several samples; (**A**) Cl 93A (×2000), (**B**) Salecan (×8000), (**C**) PC, (**D**) PCS1, (**E**) PCS2, (**F**) PCS3 (×500).

*Morphological analysis.* SEM images (Figure 2) showed that the morphology of composite xerogels were different depending on the Salecan presence and concentration used in the synthesis. The composite materials presented interconnected macropores separated by thin walls with clay aggregates distributed throughout the polymeric matrix. PMAA based composite xerogel with Cl 93A presented the largest and most uniformly distributed pores. 

According to SEM images, the presence of Salecan induced an entangled morphology containing smaller pores arranged irregularly when compared with the blank sample. As well, SEM images revealed small size pores as function of Salecan concentration. Unlike other studies that reported an increased porosity of the hydrogels when incorporating Salecan chains [33,34], in our case, the nanocomposite sample with the greatest Salecan concentration presented a more compacted morphology with the smallest size of pores. This phenomenon was caused very probably because Salecan interacted with the layered silicate through their OH groups. Both have potential to generate an advanced crosslinked network, thus influenced their distribution within the polymeric matrix. These findings correlate very well with the swelling studies, where the low values of the swelling degree were obtained for all the composite samples containing Salecan. 

Even though no clear variation could be observed from the swelling studies between the samples containing Salecan at different concentrations, SEM images showed differences in the morphologies of each sample, especially at the highest Salecan concentration. As we will notice further, the morphological features will determine doxorubicin encapsulation and release from the nanocomposite samples.

*FTIR studies*. FTIR spectra revealed the structure of the synthesized composites granting the possibility to check if the layered silicate was included in the polymeric matrix but also to follow the possible interactions occurred between the system components. The FTIR spectra of the obtained composite samples were compared with those of Cl93A and Salecan. 

**Figure 3:**
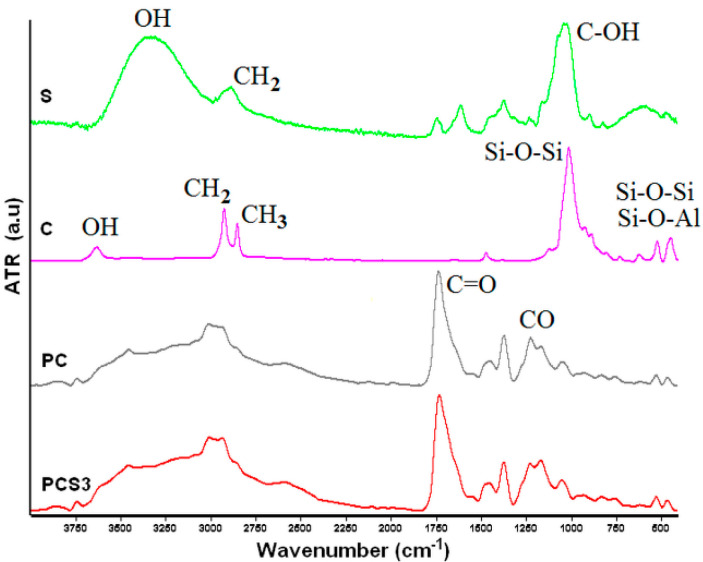
FTIR spectra of clay-C, Salecan-S and composite hydrogels without-PC Salecan and with Salecan-PCS3.

The characteristic peaks of clay were identified (Figure 3, sample C) at 1009 cm^−1^ ascribed to the Si–O–Si stretching vibration, whereas peaks at 518 cm^−1^ correspond to bending vibration of Si–O–Al and at 442 cm^−1^ to bending vibration of Si–O–Si from clay structure [35]. Peaks at 2921–2856 cm^−1^ are assigned to the asymmetric stretching vibration of methylene groups and respectively methyl, resulting from the compound used for organomodification of clay, namely quaternary ammonium salt [36]. The absorption band observed at 3634 cm^−1^ was ascribed to the hydroxyl molecule in stretching mode [37].

Salecan FTIR curves revealed at 3000–3650 cm^−1^ a broad peak due OH stretching vibrations. Stretching frequencies of C-OH were observed at 1031 cm^−1^ and of CH_2_ groups from the glucopyranose ring at 2889 cm^−1^ [33,34]. 

Composite xerogels spectra displayed in Figure 3 revealed for PCS3 sample the peaks characteristic of polymethacrylic acid, namely C=O and C-O from carboxylic groups, identified at 1733 cm^−1^ and correspondingly at 1371 cm^−1^. The specific peaks of clay were found to be shifted in the composite FTIR curves, specifically the peak from 1009 cm^−1^ shifted towards ~1047 cm^−1^ and the peaks from 442 cm^−1^ and 518 cm^−1^ shifted to 458 cm^−1^ and 523 cm^−1^. Other changes were observed in the 2750–3750 cm^−1^ range. These shifts are possible due the possible hydrogen bonding between OH located at clay edges and PMMA hydroxyl moieties [38]. 

The presence of Salecan brought modifications in the spectra of composite hydrogel. Thus, the C-OH stretching vibration from Salecan glucopyranose ring was reduced in intensity and was partly covered at 1047 cm^−1^ and 1025 cm^−1^ with Si–O–Si peak from clay due asymmetric stretching vibration, determining its shifting. Moreover, when Salecan content increased, the intensity of the specified peaks became greater. These results suggest that interaction between the OH groups of biopolymer and inorganic compound obviously took place. 

Thereby, FTIR modifications observed for the composite xerogels obtained in the presence of Salecan indicated the participation of the hydroxyl groups present in PMAA, clay and Salecan to the formation of crosslinking sites. These results demonstrated that hydrogen bonds had an essential contribution in the design of PMAA-Salecan-clay semi-interpenetrated hydrogels, each component bringing its own contribution, and thus demonstrating the achievement of an advanced crosslinked network.

**Figure 4:**
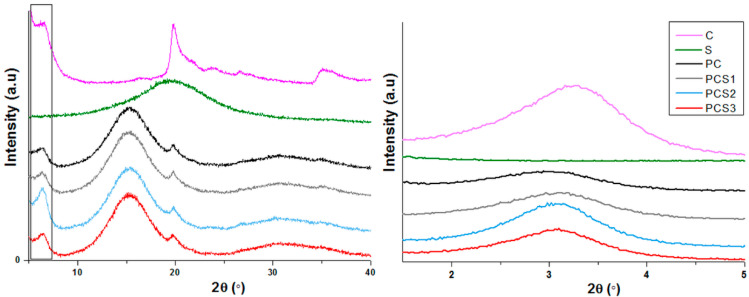
XRD patterns (wide angle—I and low angle—II) for clay, Salecan and synthesized nancomposite samples.

*XRD studies.* XRD spectra of Salecan powder showed only one peak at 2theta = 21° (WA), which was ascribed to the semi-crystalline areas of biopolymer due to hydrogen bonds of Salecan which may intra and inter-molecular interact [34]. X-Ray diffractograms obtained for clay, Salecan, composite hydrogel and Salecan composite hydrogels are presented in Figure 4.

As reported by other researchers, the loss of crystalline structure in the composite samples could be caused by electrostatic interaction occurred between monomers or their interaction with clay [34,39].

In the case of Cl 93A, XRD spectra showed the presence of the characteristic diffraction peak at 2θ ~ 3.28° which corresponds to the interlayer spacing of the crystalline structure d_001_. Specific rearrangements related to polymer/biopolymer insertion between clay layers lead to changes in the clay basal spacing values (d_001_) thus, indicating if intercalation and/or exfoliation of clay were achieved. Clay characteristic peak broadened and shifted toward lower angles in the case of composite samples thereby increased (d_001_) dimensions were registered demonstrating polymer insertion between clay layers and consequently the formation of intercalated/exfoliated composites. At lower values of Salecan concentration, the difference in peak intensity and displacement is imperceptible. While the Salecan concentration increased a slight increase of peak intensity and minor shifting toward higher values were observed. These results indicated that the presence of Salecan induced a more compacted structure.

**Figure 5:**
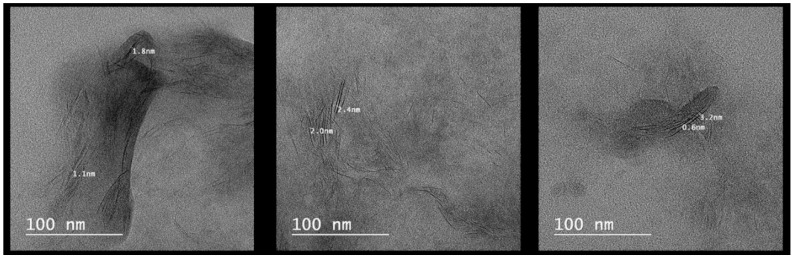
TEM images recorded on the polymethacrylic acid (PMAA)–Salecan xerogel matrix—sample PCS3.

*TEM studies.* TEM analyses can provide a qualitative understanding of dispersion of the clay inside polymer matrix by direct visualization [40]. In Figure 5, the dark lines show the clay galleries and the light region corresponds to the PMAA-Salecan matrix. TEM images recorded on the PCS3 sample evidenced intercalated but also exfoliated clay layers as a result of the loss of clay ordered stacking structure during nanocomposites synthesis. These observations correlate very well with XRD analyses which showed changes in the clay basal spacing values as a consequence of polymer insertion. 

### 3.2. Dox Loading in Nanocomposite Systems

**Figure 6:**
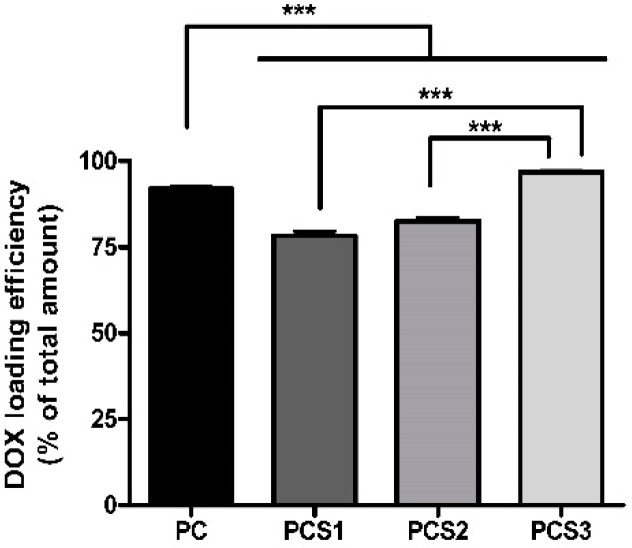
Efficiency of DOX loading into PC, PCS1, PCS2, and PCS3 composite systems. Data are presented as mean values ± SD, and significance was determined at *** *p* < 0.001 compared to PC DOX loading.

High DOX loading in the composite systems were achieved ranging from 79 to almost 97% DOX (Figure 6). The composite samples based on PMAA reached almost 92% because of the electrostatic interactions occurred between the composite hydrogels negatively charged and DOX molecules which are positively charged. The DOX efficiency of PCS1 decreased, possibly due the drop down of the swelling degree and the formation of a more compacted structure in the presence of Salecan as observed from SEM images (Figure 2). It is known that the presence of Salecan is important for the structure and dimensions of pore sizes of the resulted hydrogel [14,23,41,42]. In our case, the efficiency of DOX loading increases with the concentration of Salecan, a behavior which could be related with the decrease in pore size dimensions, captured for PCS2 and PCS3 samples. The decrease of pores size leads to an available greater specific surface of the porous hydrogel based composite structures, which favors the loading of DOX molecules.

### 3.3. DOX Release from Nanocomposites Systems

**Figure 7:**
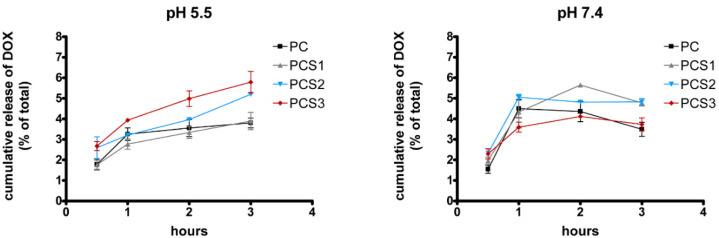
Doxorubicin (DOX) release from PC, PCS1, PCS2, and PCS3 systems at pH 5.5 and 7.4. Values are calculated based on DOX standard curve (0–400 µg/mL).

The hydrogels systems are predicted to be used in the treatment of gastro-intestinal cancer. For this purpose it was of interest to determine DOX release from these systems at pH values corresponding to the tumoral (pH 5.5) and normal environment (pH 7.4), respectively. All systems were analyzed for DOX release after 1, 2, and 3 h, for both pH value (Figure 7). The results obtained at physiologic pH after 1 h of incubation showed a quick kinetic of DOX release from all the systems. After this time point a plateau is reached for all formulations, with small differences between samples regarding the DOX release. This behavior is maintained after 24 h probably due to non-sink conditions experiments (data not shown).

Unlike physiological conditions, a more controlled release of the active compound from the systems was detected in the case of the acidic pH. In an acidic environment the carboxyl groups from PMMA structure are protonated and disrupt the electrostatic interactions between hydrogel and DOX favoring its release. However, unlike the observation of Liang et al. [23], in our study the DOX release behavior was inversely connected with the ratio swelling and pore size dimensions, and a direct correlation with the amount of Salecan was observed. Thus, PCS1 sample which contains the lowest quantity of Salecan from all systems revealed a slower release of DOX, as compared to the system without Salecan (PC). The systems containing increased amount of Salecan, PCS2, and PCS3 led to a more DOX released from the system, the highest amount being obtained for PCS3 sample after 3 h. Notably, at tumoral environment pH value the differences regarding DOX release between the tested formulations are also small.

Generally, it can be stated that at acidic pH DOX release from the nanocomposites systems is positively correlated with the concentration of Salecan in the samples, while at pH 7.4 the system which contains the lowest concentration of Salecan released the highest quantity of DOX, as compared to the other systems (Figure 7). This behavior was reported by other groups when investigated pH-sensitive composite hydrogel containing Salecan as drug carrier for DOX [14,34,42].

Taking into consideration that the differences between samples regarding DOX release are small, all hydrogel based nanocomposites have been investigated for DOX cellular up-take and cytotoxic capacity against normal and tumoral cells.

### 3.4. In Vitro Studies

**Figure 8:**
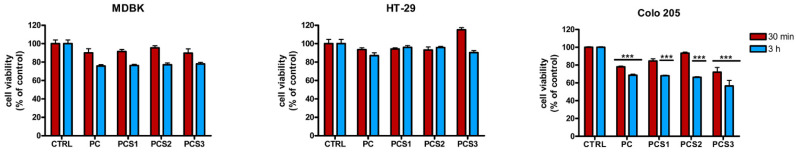
Viability of MDBK (Madin–Darby bovine kidney), HT-29 and Colo 205 cells cultivated in direct contact with nanocomposites systems (PC, PCS1, PCS2, PCS3) for 30 min and 3 h. Cell viability was expressed as percent of control (untreated cells) + standard deviation, *** *p* value < 0.001 was considered statistically significant.

*Cytotoxicity.* The effectiveness of the DOX release from different hydrogel systems (PC, PCS1, PCS2, PCS3) on normal (MDBK) and tumoral (HT-29 and Colo 205) cells was investigated at 30 min and 3 h and compared with control (untreated cells) (Figure 8). Previously we showed that biopolymer modified clays were non-toxic and had no effect on morphology and proliferation of normal (MDBK) and adenocarcinoma cells (HT-29) irrespective of time of incubation, thus system components do not interfere with nanocomposites system biocompatibility [17]. In the present work we investigated additionally on Colo 205 cells, a model for colon cancer, previously used by our group to investigate the chitosan-coated PLGA-NPs uptake and the potential use as drug delivery system in cancer therapy [43]. 

The results presented in Figure 8 revealed a time-dependent cytotoxic effect of DOX released from the systems, especially for normal and Colo 205 cell lines. Thus, in MDBK cells the viability was 90–92% at 30 min and ~80% at 3 h for all the tested systems. The cytotoxicity assay revealed a decrease (*p* < 0.001) in Colo 205 cell viability for longer period of time (3 h), with no difference between the samples detected, except PCS3 (highest concentration of Salecan) which reached a drop in tumor cell viability of ~40%. This feature may be related to the capacity of PCS3 system to release the highest amount of DOX at acidic conditions compared to the other systems analyzed (Figure 7). HT-29 cells exhibited a good viability at both 30 min and 3 h time points. 

The different effect of hydrogels systems on the two human colon cancer cell lines might be explained by (i) HT-29 cells capacity to become more resistant to chemotherapeutic drug [44] and also by (ii) increased cell surface exposure with Colo-205 metastatic cells of released DOX since they are a heterogeneous cell line exhibiting both adherent and suspended states [45].

In our experiments, performed in similar conditions, equivalent concentrations of free DOX and PCS3 released DOX revealed same inhibitory effect of cell viability in the case of tumoral Colo205 cells. This demonstrates that DOX entrapment in hydrogel nanocomposites does not affect its antitumoral activity. However, free DOX exhibited a cytotoxic effect in the case of normal MDBK cells, as opposed to DOX included in hydrogel nanocomposites (data not shown). These findings show that hydrogel based nanocomposite containing DOX, with the highest amount of Salecan (PCS3), is nontoxic on normal cells, but at the same time is efficient in decreasing the cancer cells proliferation.

*Cell proliferation and internalization*. Nanocomposites systems need to be efficient regarding the release and subsequent cellular up-take of DOX by tumoral cells. Ki-67 protein is present during all active phases of the cell cycle, but is absent in resting cells (phase G0). This makes it an excellent marker for actively proliferating cells detection when analyzing different cell populations [46]. Importantly, in proliferating cells the Ki-67 protein is present in small foci in the cytoplasm, where the sites for protein synthesis are located.

**Figure 9:**
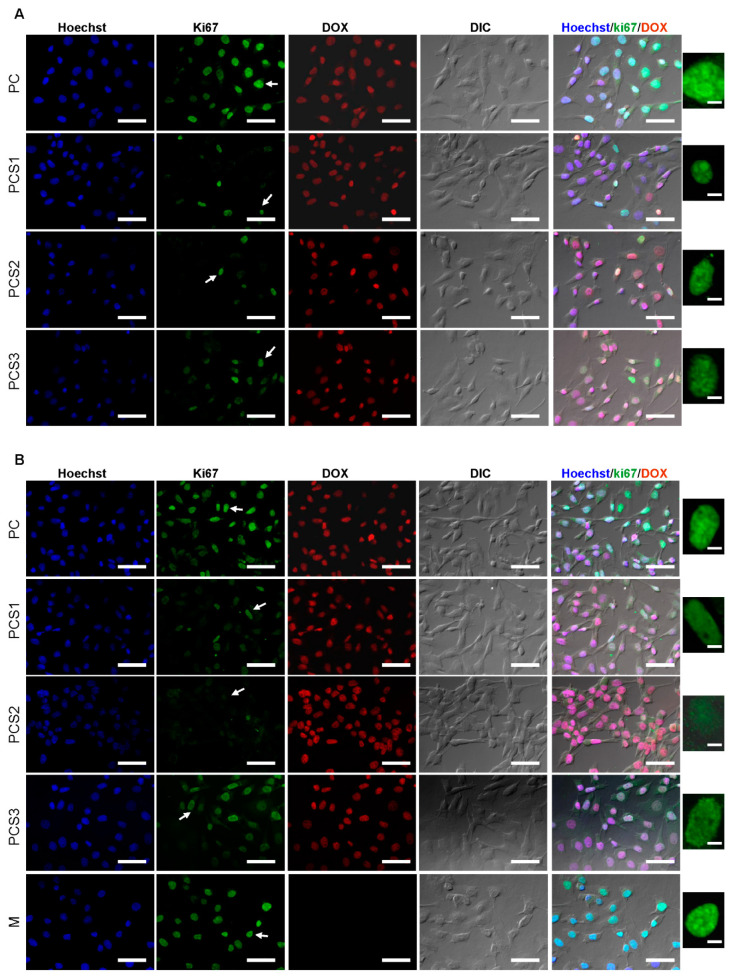

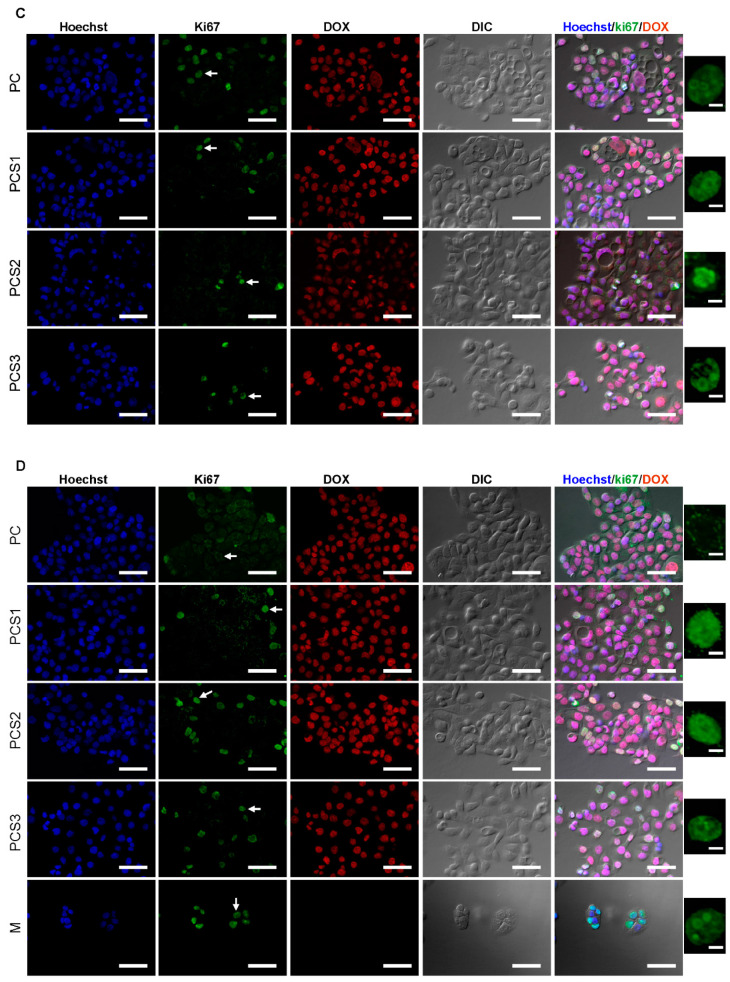

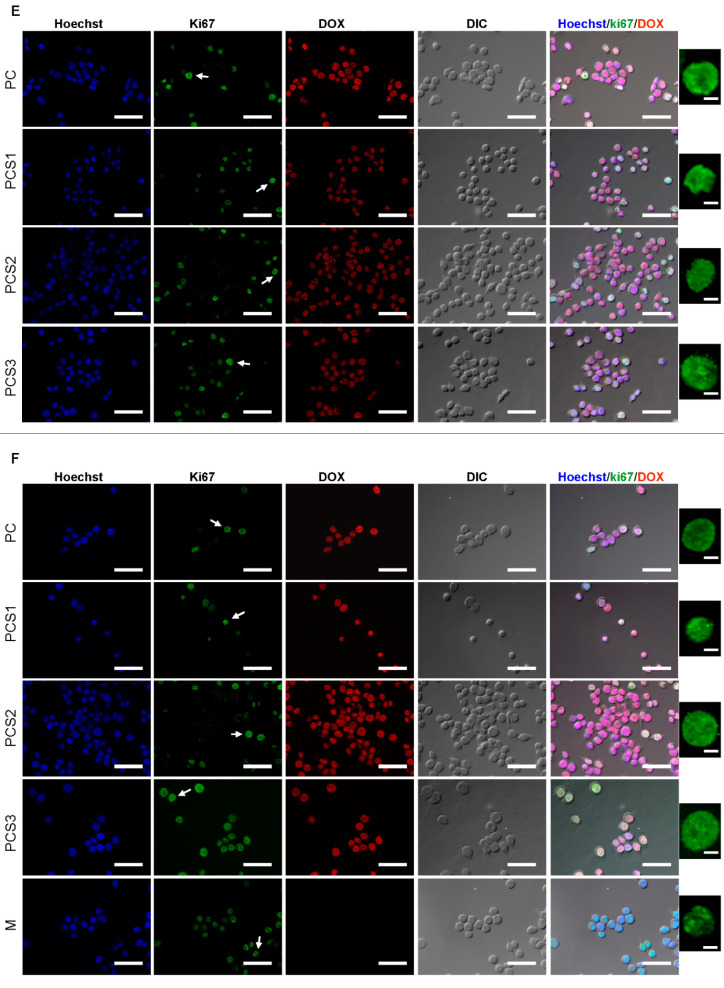
Fluorescence microscopy and differential interference contrast (DIC) micrographs of MDBK, HT-29 and Colo 205 cells after 30 min and 3 h of incubation with nanocomposites systems (MDBK (**A**) 30 min (**B**) 3 h, HT-29 (**C**) 30 min (**D**) 3 h, Colo 205 (**E**) 30 min, and (**F**) 3 h). DOX up-take by cells was recorded by DOX (red) and nucleus (blue) imaging by fluorescence microscopy. Cell proliferation and cell cycle were detected by specific labeling with Ki67 (green) of proliferating cells and visualizing with 40× objective (scale bar, 50 μm). Nuclear localization (white arrow) of Ki67 was detected in cells in different phases of the cell cycle (small pictures on the right, scale bar, 5 μm). M-cells without nanocomposites treatment.

Normal MDBK and tumoral HT-29 and Colo 205 cells were treated with the four nanocomposites systems for 30 min and 3 h at 37 °C and the compound internalized was visualized by fluorescent microscopy. In Figure 9 are depicted fluorescent and differential interference contrast (DIC) microscopy images. For all cell lines, the red fluorescence emitted by DOX can be observed overlapping the nuclei labeled with Hoechst (blue), suggesting DOX nuclear localization. This observation was recorded after 30 min of treatment. An enhancement of red fluorescence intensity at the intranuclear level was observed in the case of cells treated for 3 h with nanocomposites systems. Moreover, an accumulation of the antitumor compound at the nuclear level was detected in the case of tumoral cells HT-29 and Colo 205. 

The treatment with nanocomplex systems led to a mild decrease of the number of proliferating cells in the case of MDBK cells, with the most significant results in the PCS1 and PCS2 systems after only 30 min of treatment (Figure 9A,B). The other two systems, PC and PCS3, have a less pronounced anti-proliferative effect, since in these samples a growing number of Ki67 positive cells were observed. After 3 h of treatment with PCS2 system, an increased anti-proliferative effect was detected (Figure 9B).

Interestingly, although HT-29 cell line was found to internalize high amount of Dox at both time points (Figure 9C,D), the antiproliferative effect of the compound released from the hydrogel systems is less evident. These results are in accordance with the absence of cytotoxic effect previously depicted in Figure 8. 

All these observations were in agreement with previous studies performed on human intestinal epithelial HT-29 cell line, which revealed that Dox accumulates in HT-29 cells as early as 1 h after incubation, but despite extensive incubation up to 24 h, a decrease in viability was not detected [47].

Fluorescence microscopy investigations revealed a decrease in the number of Colo 205 tumor cells—a cell line of adenocarcinoma derived from metastatic situs—in the microscopy slides at 3 h compared to 30 min of treatment. The treatment with PC and PCS1 systems led to a high anti-proliferative effect, as shown by a reduced number of Ki67+ cells (Figure 9E,F). Additionally, PCS2 system revealed an increased cytotoxic effect after 3 h of treatment on Colo 205 cell line. Among the tested systems, PCS3 led to a higher anti-proliferative capacity which correlates with an increase nuclear DOX distribution and cytotoxic effect.

**Figure 10:**
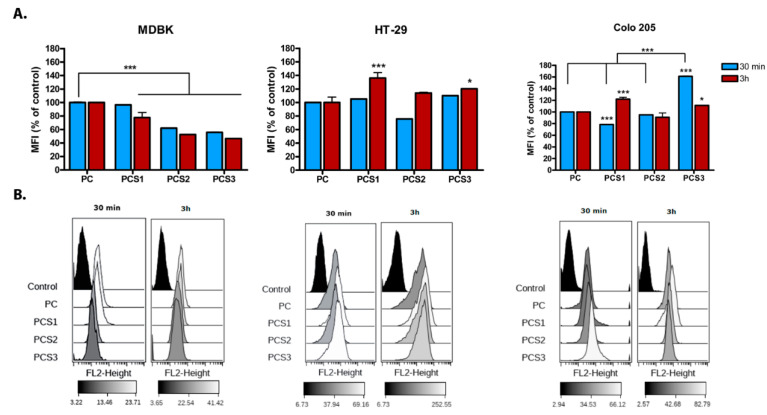
Flow cytometry for DOX released from nanocomposites systems and internalized into MDBK HT-29 and Colo 205 cells, after 30 min and 3 h of treatment. (**A**) Graphical representation; Mean fluorescent intensity (MFI) for PC control was set to 100%. (**B**) Histograms overlay and gray scale gradient of fluorescence representation using Cytobank; control-untreated cells. *** *p* value < 0.001; * *p* value < 0.05.

DOX internalization into cells was confirmed by flow cytometry experiments (Figure 10A,B). The recordings of mean fluorescence intensity of DOX measured by FACS and presented in the histograms showed that, in the case of MDBK cells, DOX released from all systems containing Salecan (PCS1, PCS2, and PCS3) exhibits a slower kinetic of internalization, as compared with the system without Salecan (PC), for both time of incubation. The up-take of DOX decreases with the increase of Salecan amount in systems composition. This behavior was accentuated in the case of PCS2 and PCS3 samples, where the intensity of DOX florescence was 50% reduced as compared to PC. Generally, for all hydrogel systems, the Dox internalization is dose-dependent of Salecan and correlates with the good viability observed in the case of normal cells (Figure 8).

In the case of HT-29 tumor cell line, a rapid kinetic of DOX internalization released from the systems was observed (Figure 10A) and the amount of DOX internalized is significantly increased than those obtained for MDBK cells, especially after 3 h of treatment. These results can be better evaluated in Figure 10B where the data for Dox up-take in HT-29 are depicted as histograms. Thus, it is showed that the highest amount of DOX internalized at 3 h is in HT-29 cell line (gray scale gradient: 252 in HT-29 versus 82 in Colo 205 versus 41 in MDBK) from all the cell lines tested.

Interestingly, the rate of DOX up-take slightly decreases with the amount of Salecan, a feature observed at 3 h time point. This observation concerns especially PCS1 (*p* < 0.001), where an accentuated increase of DOX internalized by HT-29 tumoral cells was detected. Although HT-29 internalized the highest amount of Dox released from the systems, its viability is not affected. These results correlate with a low cytotoxicity observed in Figure 8, a reduced anti-proliferative effect in Figure 9C,D and its reported chemoresistance to DOX [44].

Unlike HT-29 cells, a rapid cellular DOX up-take by Colo 205 cells and a direct correlation with Salecan amount was observed for short period of time (30 min) the highest amount of compound being recorded in the case of PCS3 samples (high concentration of Salecan), as compared to PC control (no Salecan) (Figure 10A). As it was shown, PCS3 has also the highest capacity to release the quantity of DOX at acidic pH and to decrease the viability (under 60%) of tumor Colo 205 cells. These suggest an important role of Salecan in the release of the active compound into cells and are in agreement with the cytotoxicity results revealing an inverse correlation with the increase in Salecan concentration in the samples. The values of DOX fluorescence intensity recorded after 3 h of treatment showed a slightly increase in cell internalization for PCS1 compared to the other systems with or without Salecan. Interestingly, in the case of PCS3 sample a decrease in DOX internalization in Colo 205 cells from 160% at 30 min to 100% versus PC control at 3 h was observed. These data can be explained by the decrease in cell viability, as DOX intracellular quantification by flow cytometry was performed only with intact cells. All these results are better depicted in Figure 10B as histograms. Thus, it is showed that higher amount of DOX is internalized at 3 h in Colo205 cell line as compared to normal MDBK cell line (82 in Colo205 versus 41 in MDBK). Moreover, unlike HT-29 cells, for this cell line a cytotoxic effect was obtained (Figure 8).

The methods used in the present study underscore the evidence that all cell lines employed (normal or tumoral) internalized the active compound DOX released from nanocomposites systems with a nuclear localization. However, it is important to mention the difference observed between DOX internalization processes in the two tumor cell lines. Thus, for HT-29 cells the highest amount of DOX was internalized after 3 h, the compound being released by the system with the lowest Salecan (PCS1) in composition. On the contrary, in Colo 205 cells, DOX internalization was higher at 30 min and correlates directly with the amount of Salecan in the tested systems (maximum for PCS3). These can be partially explained by a larger cell surface exposed in direct contact with nanocomposites systems, due to the morphological features characteristic for Colo 205 metastatic cell line, such as low adherent, partial suspension cell. 

## 4. Conclusions

In this study, novel nanocomposites systems based on semi synthetic networks in the presence of organomodified clay were successfully prepared and investigated. It was found that Salecan concentration determined the swelling behavior, the structural parameters and the morphological features of the nanocomposite systems. The results underline the important role played by hydrogen bonds design of PMAA-Salecan-clay hydrogels, each component bringing its own contribution, and thus demonstrating the achievement of an advanced crosslinked network and more compacted hydrogel nanocomposite morphology.

The efficiency of DOX loading and its release from the synthesized nanocomposites systems, the cellular up-take by normal and tumor cells and the effect of the drug on cell proliferation were further investigated. DOX loading efficiency was found to increase with the concentration of Salecan due to the decrease of pores size which favored DOX molecules entrapment. At acidic conditions, DOX release was positively correlated with the concentration of Salecan in the samples, while at physiological conditions (pH 7.4) the system which contained the lowest concentration of Salecan released the highest quantity of DOX, as compared to the other systems. Toxicity assays confirmed that the blank sample (PC) had negligible toxicity to normal MDBK cells whereas the DOX-loaded Salecan nanocomposites (PCS1, PCS2, PCS3) were cytotoxic after 3 h, especially for Colo 205 cell line where the system containing the highest amount of Salecan (PCS3) determined a decreased by 40% of the cell viability.

The experiments of intracellular DOX localization by fluorescence microscopy correlate with the results obtained by quantification of the chemotherapeutic compound using flow cytometry. The data revealed different behaviors of the two tumor cell lines, HT-29 cells being more resistant to DOX internalization and its cytotoxic effect. In the case of Colo 205 cells, it was found that an increase in Salecan composition in nanocomposites system can promote an antiproliferative effect on the tumor cell line. PCS3 system which contains the highest amount of Salecan was internalized and induced a high cytotoxic effect (up to 40%) in Colo 205 cells, being at the same time well tolerated by normal MDBK cells. This effect was correlated with the lowest pore size distribution leading to highest available specific surface area and entrapped amount of DOX which was further released from the nanocomposite sample.

Corroborating all the data it can be suggested that the synthesized nanocomposites with Salecan and clay could be good candidates as vehicles for chemotherapeutic agents.

## Figures and Tables

**Table 1:** Nomenclature of the synthesized samples.

Sample Name	Salecan (g)	Cloisite 93A (g) *
PC	-	0.6
PCS1	0.06	0.6
PCS2	0.24	0.6
PCS3	0.48	0.6

* Cloisite 93A amount was kept constant as 10% *w/v* in monomer (methacrylic acid).

## Patents

Ianchis: R.; Gifu, I.C.; Ninciuleanu, C.M.; Alexandrescu, E.; Petcu, C.; Nistor, L.C.; Niţu, S. Compositions and Synthesis Procedures of Hydrogel Clay Nanocomposites. Patent request No. 12 133753-A2, 30 December 2019.
